# A Prospective Study of the Prevalence of Tuberculosis and Bacteraemia in Bangladeshi Children with Severe Malnutrition and Pneumonia Including an Evaluation of Xpert MTB/RIF Assay

**DOI:** 10.1371/journal.pone.0093776

**Published:** 2014-04-02

**Authors:** Mohammod Jobayer Chisti, Stephen M. Graham, Trevor Duke, Tahmeed Ahmed, Hasan Ashraf, Abu Syed Golam Faruque, Sophie La Vincente, Sayera Banu, Rubhana Raqib, Mohammed Abdus Salam

**Affiliations:** 1 International Centre for Diarrhoeal Disease Research, Bangladesh, Dhaka, Bangladesh; 2 Centre for International Child Health, The University of Melbourne Department of Paediatrics and Murdoch Childrens Research Institute, Royal Children’s Hospital, Melbourne, Australia; 3 International Union Against Tuberculosis and Lung Disease, Paris, France; University of Cape Town, South Africa

## Abstract

**Background:**

Severe malnutrition is a risk factor for pneumonia due to a wide range of pathogens but aetiological data are limited and the role of *Mycobacterium tuberculosis* is uncertain.

**Methods:**

We prospectively investigated severely malnourished young children (<5 years) with radiological pneumonia admitted over a 15-month period. Investigations included blood culture, sputa for microscopy and mycobacterial culture. Xpert MTB/RIF assay was introduced during the study. Study children were followed for 12 weeks following their discharge from the hospital.

**Results:**

405 eligible children were enrolled, with a median age of 10 months. Bacterial pathogens were isolated from blood culture in 18 (4.4%) children, of which 72% were Gram negatives. Tuberculosis was confirmed microbiologically in 7% (27/396) of children that provided sputum - 10 by culture, 21 by Xpert MTB/RIF assay, and 4 by both tests. The diagnostic yield from induced sputum was 6% compared to 3.5% from gastric aspirate. Sixty (16%) additional children had tuberculosis diagnosed clinically that was not microbiologically confirmed. Most confirmed tuberculosis cases did not have a positive contact history or positive tuberculin test. The sensitivity and specificity of Xpert MTB/RIF assay compared to culture was 67% (95% CI: 24–94) and 92% (95% CI: 87–95) respectively. Overall case-fatality rate was 17% and half of the deaths occurred in home following discharge from the hospital.

**Conclusion and Significance:**

TB was common in severely malnourished Bangladeshi children with pneumonia. X-pert MTB/RIF assay provided higher case detection rate compared to sputum microscopy and culture. The high mortality among the study children underscores the need for further research aimed at improved case detection and management for better outcomes.

## Introduction

Pneumonia is the leading cause of childhood mortality globally [1], and malnutrition is a recognized risk for pneumonia and death [2]. A recent systematic review reported that the range of bacterial pathogens that cause pneumonia in children with severe malnutrition is different to those that cause pneumonia in well nourished children, and that Gram negative bacteria are predominant causes [3]. *Mycobacterium tuberculosis* is also believed to be an important cause of pneumonia in severely malnourished children, but there are surprisingly few data [3], [4], [5].

Tuberculosis (TB) is a preventable and treatable disease that can cause severe malnutrition in children, either as a primary or as an associated cause. The World Health Organization (WHO), based on vital registration data, estimated that TB caused 74,000 deaths in HIV-uninfected children globally in 2012 [6]. However, the actual contribution of TB to global child mortality is likely to be higher for two main reasons. First, this estimate does not include HIV-infected children with TB as these deaths are classified as deaths due to HIV [6]. Secondly, in endemic settings TB is commonly found in children dying with pneumonia, reported by clinical and autopsy studies in settings with high child mortality [7], [8], [9], [10].

An important contributor to uncertainties regarding prevalence of TB in severely malnourished young children is related to the challenge in confirming a diagnosis. The difficulties relate to obtaining high quality specimens, the paucibacillary nature of the disease in young children, and lack of mycobacterial culture facilities in settings where TB and malnutrition are endemic - even when available the long time delay in getting results of culture and susceptibility. The Xpert MTB/RIF assay (Cepheid, Sunnyvale, CA, USA) is a new, rapid diagnostic test for the detection of *M tuberculosis* and rifampicin resistance that is demonstrating encouraging results in the diagnosis of pulmonary TB in children using their respiratory samples. Studies in children have consistently demonstrated its greater sensitivity than smear microscopy, and additionally in providing a more rapid result than culture [11], [12], [13], [14].

The aim of our study was to determine the prevalence of bacteraemia and TB in severely malnourished children with pneumonia; compare the sensitivity and specificity of the Xpert MTB/RIF with that of mycobacterial culture in sputum samples; and to compare the TB diagnosis from induced sputum versus gastric lavage.

## Methods

### Ethics Statement

The study (protocol number: PR-10067) was approved by the Research Review Committee and the Ethical Review Committee (ERC) of the International Centre for Diarrhoeal Disease Research, Bangladesh (icddr, b). Written informed consent was obtained from parents or attending guardians of all the participating children.

### Study Design and Participants

This was a prospective, descriptive study that recruited consecutive severely malnourished children of either sex, younger than 5 years, who were admitted to inpatient wards of the Dhaka Hospital of icddr, b from April 2011 through June 2012 with respiratory symptoms (cough and/or respiratory distress) and radiological pneumonia. Children whose parents/attending guardians did not give consent were not included in the study.

### Study Setting

The Dhaka Hospital of icddr, b provides ambulatory and inpatient care to around 120,000 children annually, mostly live in the poor communities in urban or periurban Dhaka. The reported incidence of all TB cases in Bangladesh was 225 per 100,000 population in 2011 with an estimated case-detection rate of just 45% [6]. In 2011, the under 5 mortality rate was 48 per 1,000 live births and 10% of children were severely underweight [15]. Neonatal BCG coverage was 95%, and the prevalence of HIV among adults and young people was <0.1%.

### Clinical Procedures

Study children were managed according to established clinical guidelines of the hospital, including rehydration, nutritional rehabilitation, and management of pneumonia and hypoxaemia (defined as SpO_2_<90% as study site is at sea level) [16], [17]. Clinical and demographic data were collected using a case record form designed for this study, pre-tested and finalised. Chest radiography was done for all study children, following the hospital’s standard of care, and other investigations included a tuberculin skin test (TST), a blood culture, and microscopy and culture of gastric lavage and induced sputum (a single sample of each from each child) as previously described [18].

A standard procedure of TST was followed and induration measured at 72 hours [19]. Both sputum sampling procedures were performed after the children had fasted for 4 hours. Gastric lavage fluid was collected early in the morning before the children were ambulant, using an appropriate sized nasogastric tube introduced in the previous night. Samples were collected from gastric lavage and induced sputum following standard protocols, into sterile tubes and immediately sent to the TB laboratory for processing [19], [20]. Gastric lavage is an established procedure at icddr, b while sputum induction was introduced for this study following training under a concurrent study that examined the aetiology in child pneumonia [20].

Children with a diagnosis of pneumonia or TB were treated according to Bangladesh’s national guidelines. Children with severe malnutrition and radiological pneumonia (defined below) were treated with parenteral ampicillin and gentamicin as the first-line treatment, which were changed to ceftriaxone and levofloxacin for children not responding to the first line therapy. Children with a diagnosis of TB (defined below) were treated with the standard first-line regimen of 2 months of daily rifampicin, isoniazid and pyrazinamide, followed by 4 months of daily rifampicin and isoniazid per national guidelines [21]. All children were followed-up for 12 weeks following their discharge from the hospital; those treated for TB were followed for a total of 6 months.

### Laboratory Procedures

Sputum samples were decontaminated according to the standard Petroffs’ method in the tuberculosis laboratory of icddr, b as described earlier [22]. Acid fast bacilli (AFB) were detected by light microscopy on sputum smear following Ziehl-Neelsen staining and decontaminated samples were inoculated on Löwenstein-Jensen slants for mycobacterial culture as previously described [22].

The Xpert MTB/RIF assay became available during the study period in November 2011 and this investigation was incorporated into the study following approval of the amended protocol by the ERC. The gastric lavage sample was split into two separate sterile containers, and two sterile mucous extractors were used for sample collection following sputum induction. In each case, one sample was for smear microscopy and mycobacterial culture, and the other was for analysis by real-time PCR using the Xpert MTB/RIF assay following the standardized procedures [12].

Drug susceptibility testing (DST) was done for all positive cultures. In each lot of DST, a strain of H37Rv was used as the sensitive control strain and a known MDR strain was used as the resistant control strains. Mycobacterial species were identified by molecular testing as described earlier [23]. Blood cultures were processed using the BACTEC system (BioMerieux, Marcy L’Etoile, France) as previously described [24].

### Definitions

- Severe malnutrition: severe wasting [Z score for weight for height <−3 of the WHO median] or severe under-nutrition [Z score for weight for age <−4 of the WHO median], or nutritional edema.
- Radiological pneumonia: the presence of end-point consolidation or other (non-end-point) infiltrate in lungs according to the WHO radiological classification of pneumonia [25] and confirmed independently by a radiologist and pediatrician at the study site.
- Positive TST: transverse diameter of induration was ≥5 mm irrespective of BCG status. Note that all children were severely malnourished.
- Classification of TB diagnosis: All children had typical TB-related symptoms at entry point and parenchymal abnormalities on chest radiograph as per eligibility for inclusion in the study. Therefore, for purposes of this study,
  - “confirmed TB” means identification of *M tuberculosis* by culture or by Xpert MTB/RIF assay on any of the test specimens.
  - “not confirmed TB” means when a clinical diagnosis was not confirmed microbiologically with supportive evidence such as positive TST or a positive contact history or when there was no symptomatic improvement of bacterial pneumonia or severe malnutrition following therapy.
  - “not TB” means all other children who were enrolled in the study and had completed the assessments (i.e. sputum sample collected and TST performed and interpreted). The children who did were not fully assessed due to their deaths or had left the hospital before collection of sputum or before reading TST results were not included in categorization of TB diagnosis.

### Data Analysis

Case Report Forms were developed, pretested, and finalized for data acquisition. Data were entered using SPSS for Windows (version 17.0; SPSS Inc, Chicago) and Epi Info (version 6.0, USD, Stone Mountain, GA). We evaluated the frequencies of bacterial isolates from blood culture and of *M.tuberculosis* by culture and Xpert MTB/RIF from sputum. For continuous data, mean (standard deviation) and median (inter-quartile range) are reported for symmetrically and asymmetrically distributed data respectively, with comparisons by t test and Mann-Whitney test. For categorical data, differences in proportions are compared by the Chi-square test. Strength of association was determined by calculating odds ratios (OR) and 95% confidence intervals (CI). We evaluated the sensitivity and specificity of Xpert MTB/RIF compared to culture.

## Results

**Figure 1:**
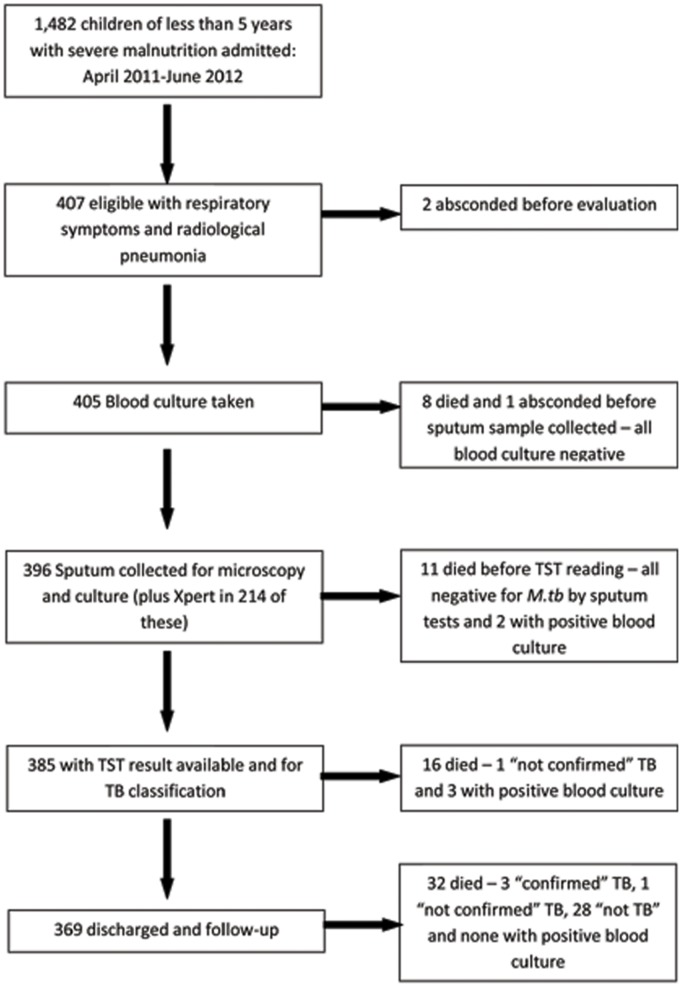
Flowchart of enrolment, investigations and outcome.

1,482 severely malnourished children of either sex, younger than 5 years, were admitted to the Dhaka Hospital during a 15-month period (April 2011–June 2012). Of them, 405 had respiratory symptoms with radiological pneumonia, and were included in the study. The median (IQR) age was 10 (5, 18) months. Not all children were fully investigated and thus not clinically classified mainly due to deaths before completion of all investigations, or left hospital before such assessments could be made. Figure 1 provides information on the children, the investigations done, and the outcome.

### Bacteraemia

A bacterial pathogen was isolated from blood in 4% (18/405) of children, and 72% (13/18) of them were Gram negative bacteria (Table 1). Two children had 2 bacterial pathogens isolated from the same blood culture. An additional 22 (5%) isolates were considered contaminants. Antibiotic usage prior to admission was reported for 12% of all the study children and for 17% (3/18) of the children with bacteremia.

**Table 1:** Bacterial and mycobacterial isolates in study children.

	Number of patients with results	Positive (%)
**Bacterial isolates from blood culture**		
**Pathogens**	405	18 (4)
*Streptococcus pneumonia*		4
*Klebsiella* species		2
*Haemophilus influenza*		2
*Salmonella typhi*		2
*Acinetobacter*		2
*Staphylococcus aureus*		1
*Salmonella enteritidis*		1
*Pseudomonas species*		1
*Enterobacter species*		1
Polymicrobial*		2
**Contaminants**	405	22 (5)
Coagulase negative *staphylococci*		21
*Staphylococcus haemolyticus*		1
**Mycobacterial isolates from sputum**		
*Mycobacterium tuberculosis*	396	27 (6.8)
Culture	396	10 (3)
- Xpert MTB/RIF positive	4	4
- Xpert MTB/RIF negative	2	2
- Xpert MTB/RIF not done	182	4
Xpert MTB/RIF	214	21 (10)
- culture negative	214	17
- culture positive	214	4
*Mycobacterium fortuitum*	396	4 (1)

*E.coli + *Enterococcus* and *Klebsiella* + *Pseudomonas*.

### Tuberculosis Diagnosis

Sputum was examined by smear microscopy and culture alone in 191 children. X-pert MTB/RIF assay was introduced later, during the conduct of the study, and was done in an additional 214 children (Tables 1 and 2). TB was microbiologically confirmed on either or both the specimens in 7% (27) children - 10 by culture, an additional 17 by Xpert MTB/RIF, and 4 (16%) by both the tests i.e. a total of 27 children. Six children (24%) were AFB positive on smear microscopy but two (8%) were not culture confirmed and thus not regarded as TB cases. Among 21 children positive by X-pert MTB/RIF alone, only one was AFB positive by smear microscopy. An additional four children were culture-positive for *Mycobacterium fortuitum* from gastric lavage samples, and an additional one positive from induced sputum. None of the confirmed TB cases was bacteraemic.

**Table 2:** Results of smear and Xpert in relation to mycobacterial culture results.

	Culture positive	Culture negative	Total
	N = 10	N = 386	N = 396
**Xpert Positive**	4	17	21
Smear Positive	1	0	1
Smear Negative	3	17	20
**Xpert Negative**	2	191	193
Smear Positive	0	0	0
Smear Negative	2	191	193
**Xpert not done**	4	178	182
Smear Positive	3	2	5
Smear Negative	1	176	177

Taking culture confirmed TB as the ‘gold standard’, the sensitivity of Xpert MTB/RIF was 67% (95% CI: 24–94) and specificity was 92% (95% CI: 87–95). The ability to diagnose *M.tuberculosis* from culture of gastric lavage and induced sputum, and by Xpert MTB/RIF are shown in Table 3. The yield did not differ significantly between the methods of specimen collection. Only one patient was positive by both the diagnostic methods and on both the specimens. Two children were diagnosed with multi-drug resistant TB (MDR TB) by culture and DST - resistant to rifampicin, isoniazid, ethambutol and pyrazinamide. For both the children, culture was positive on both the specimens, while Xpert MTB/RIF detected rifampicin resistance only from the gastric lavage specimen. These children were referred to the national TB centre for further management and treatment. None of the Xpert positive, culture negative results identified rifampicin resistance.

**Table 3:** Comparison of diagnostic yield from study patients according to sampling method and laboratory method.

Diagnosis	Induced sputum (IS)	Gastric lavage (GL)	IS and GL positive in same patient	Test of significance comparing IS to GL
	Number positive/number of samples (%)			OR (95% CI); p value
Culture	10/394 (2.5%)	6/396 (1.5%)	6/394 (1.5)	1.69 (0.56–5.29); 0.4
Xpert MTB/RIF	16/211 (7.6%)	11/214 (5.1%)	6/211 (2.8)	1.51 (0.65–3.59); 0.4
All confirmed TB	24/394 (6.1%)	14/396 (3.5%)	11/394 (2.8)	1.77 (0.86–3.66); 0.13

OR = Odds ratio; CI = Confidence interval.

A diagnostic classification of TB could be made in 385/405 of the enrolled children (Figure 1). There were 60 (16%) children with a clinical diagnosis of “non-confirmed” TB, and thus TB was clinically or microbiologically diagnosed in 87/405 (23%) children for whom all needed data were available. Table 4 lists the clinical characteristics and outcomes of the groups by TB status i.e. ‘confirmed’ and ‘no-TB’. Confirmed TB cases were significantly more likely to have a history of contact or have a positive TST than those classified as “not TB”. Of the confirmed TB cases, only 19% had a reported history of contact and only 22% were TST positive.

**Table 4:** Characteristics of study children on admission according to aetiological diagnosis.

Parameters	Classification of TB			P value – compare Confirmed TB to Not TB	Bacteraemia¶		P value – compare confirmed TB to bacteraemia
	Confirmed	Not confirmed¶	Not TB¶				
	n = 27	n = 60	n = 298		n = 18		
	n (%)	n (%)	n (%)		n (%)		
*Characteristics on admission*							
Age in months (median, IQR)	14.0 (6.0, 24.0)	11.0 (6.0, 16.0)	9.0 (4.9, 18)	0.12	5.5 (4.0, 15.5)		0.16
Male	18 (67)	35 (58)	166 (56)	0.37	10 (56)		0.66
Low socio-economic status*	24 (89)	50 (83)	250 (84)	0.78	14 (78)		0.41
Reported household TB contact	5 (19)	8 (13)	1 (0.3)	<0.01	0		0.07
BCG immunization	23 (85)	56 (93)	252 (85)	1.0	13 (72)		0.45
Cough present	25 (93)	63 (93)	254 (85)	0.39	17 (94)		1.0
Duration of cough in days (median, IQR)	7 (3.5, 8.0)	5 (4, 7)	5 (3, 7)	0.05	5 (3.5,6.5)		0.08
Weight for age Z score (mean ± SD)	−5.0±1.0	−4.8±1.2	−5.0±1.6	0.92	−5.3±1.6		0.57
Weight for height Z score (mean ± SD)	−3.7±1.6	−3.9±1.3	−3.8±1.5	0.82	−4.6±1.5		0.06
MUAC in centimetres (mean ± SD)	10.5±1.5	10.9±1.1	10.4±1.3	0.11	9.9±1.3		0.22
Axillary temperature in degrees Celsius (mean ± SD)	37.4±0.9	37.6±1.0	37.6±0.9	0.38	37.6±1.1		0.22
Respiratory rate in breaths per minute (mean ± SD)	43±11	48±12	48±11	0.02	50±10		0.01
Tachypnoea**	4 (17)	31 (52)	125 (45)	0.01	6 (33)		0.17
Chest indrawing	8 (30)	27 (45)	122 (41)	0.34	8 (44)		0.48
Hypoxemia*** (mean ± SD)	1 (4)	3 (5)	30 (10)	0.49	3 (17)		0.29
*Results of investigations*							
Bacterial isolates in blood	0 (0)	6 (10)	12 (4)	0.61	18 (100)	-	
Tuberculin skin test positive	6 (22)	56 (93)	3 (1)	<0.01	2 (11)	0.69	
CXR abnormalities							
End-point consolidation****	1 (4)	2 (3)	21 (7)	1.0	2 (11)	0.55	
Other (non-end-point) parenchymal infiltrate****	26 (96)	58 (97)	277 (93)	1.0	16 (89)	0.55	
Miliary pattern	1	0	0	0.08	0	0.08	
Pleural effusion	1	0	0	0.08	0	0.08	
*Outcome data* §							
Inpatient deaths	0 (0)	1 (1.7)	15 (5)§	0.2	5 (28)§	<0.01	
Post-discharge deaths	3 (11)	1/59 (1.7)	28/266 (11)	1.0	0/13 (0)	0.54	

¶ Note that children with bacteraemia are included in those classified as “non-confirmed” TB and “not” TB.

*defined as monthly family income of less than US$125.

**according to age: 2–11 months >50 breaths per minute; 12–59 months >40 breaths per minute.

***Oxygen saturation in air less than 90% (at sea level).

****according to WHO classification for radiographic pneumonia.

§ There were 19 inpatient deaths that occurred before all tests were completed and so are not included in classification of TB. Two of these early deaths were in children with bacteraemia (both *Acinetobacter*) and both were sputum smear and culture negative. The other 3 deaths in children with bacteremia are included in children also classified as “not TB”.

### Study Outcomes

Outcomes of the study children by their diagnosis have been presented in Table 3 and in Figure 1. Of the enrolled children, 9% (35/405) died in hospital and another 9% (32/369) of the children discharged from the hospital died in their homes during the follow-up period. Five (28%) of the bacteremia cases died in hospital but none during follow-up. The children with TB diagnosis were followed for 6 months. Of those with confirmed TB none died in hospital but 11% (3/27) died in home while receiving anti-TB treatment. Inpatient deaths was significantly higher for children with bacteraemia compared to TB children who did not have bacteraemia (p<0.001), but the post-discharge deaths was higher (not statistically significantly) among TB children. Completion of anti-TB treatment could be recorded for 60% (16/27) of the “confirmed” TB and 83% (50/60) of “non-confirmed (probable cases) TB’ cases. The two children with MDR TB responded well to initial treatment but their final outcome was not known. The children with positive *Mycobacterium fortuitum* culture were not treated prior to the culture result, were doing well clinically without any treatment, and were all well at 3-months’ follow-up.

## Discussion

This study provides original data of the prevalence of TB in severely malnourished children with pneumonia living in a TB endemic setting. There are few such data from TB endemic settings and very limited data from the Asian region - surprising given the strong clinical and epidemiological association between severe malnutrition and TB in children [4].

Our study also provides original data on the performance of Xpert MTB/RIF assay in our contexts, which provided diagnostic assistance in children with a negative sputum culture. Its specificity was lower than reported in previous studies, which may reflect the limitations of the solid culture methodology used in our study, the paucibacillary nature of TB disease in young children, and/or the quality of sputum samples collected from such sick, young children. Previous studies of Xpert included a wider age range of children (including children of 5 years and older) and compared Xpert MTB/RIF to liquid culture [12], [13], [14]. Previous studies have also noted that diagnostic yield was age-related. The sensitivity observed in our study was within the range reported by earlier studies but our range was very wide due to the small number of culture positive cases for comparison. The diagnostic yield of Xpert in our study was higher than for smear microscopy, which is consistent with earlier observations. Xpert MTB/RIF successfully detected two children in our study with MDR TB, which is consistent with the observation by Nicol et al. [12], however, both were from gastric lavage specimens while the culture was positive for both gastric lavage and induced sputum.

Our study has a number of important limitations that are inherent to studies of aetiology of respiratory disease in young children. Blood culture is recognized to have low sensitivity for determining bacterial causes of pneumonia [26]. Isolates of coagulase-negative *Staphylococcus* were considered contaminants but that could have a pathogen in some children. The true sensitivity of blood culture for bacterial pneumonia in this population is not known but is likely to be less than 20%. The true sensitivity of confirm TB by culture and Xpert assay is not known, especially in severely malnourished young children, such as children in our study. Finally, HIV testing was not performed despite its importance, at last as co-infection, in severely malnourished children; however, HIV prevalence is very low in Bangladesh, especially in children [27].

We recruited only severely malnourished children with evidence of lower respiratory tract infection diagnosed on the basis of a combination of respiratory symptoms with radiographic evidence of parenchymal lung disease, rather than using the WHO clinical classification of pneumonia that relies on respiratory rate and chest indrawing [17]. It is recognized that clinical and radiological determinants of pneumonia in children with severe malnutrition are different to those without severe malnutrition, and that they are not as well defined or validated for children with severe malnutrition. Most of the children in this study did not have fast breathing or chest indrawing, including those with bacteraemia, and it is difficult to distinguish pneumonia from septicaemia without an identifiable infections focus in such children. Severely malnourished children are profoundly immunosuppressed and unlike their healthy counterparts do not mount vigorous inflammatory response to infection [28]. This is likely to influence clinical and radiographic manifestation of disease such as bacterial pneumonia or TB.

The inpatient mortality was high, especially among those with bacteraemia, which is similar to previous studies in malnourished children [2], [16]. The deaths was associated with wide range of bacterial isolates from blood culture with predominance of Gram negatives, which is also consistent with previous observations [2], [4], [24], [29]. We also noted post-discharge mortality, with rates similar to that of the inpatient deaths. Usually, full clinical and nutritional recovery cannot be achieved during hospitalization of children in busy hospital set ups n developing countries. Consequently, those surviving during the hospitalization period may return home in a vulnerable state, and there is additional risk of acquiring nosocomial infections while in the hospital that may not be apparent at discharge. The role of antibiotic prophylaxis such as cotrimoxazole preventive therapy in this context is currently being evaluated in a randomized placebo-controlled trial in Kenyan children with severe malnutrition [30].

We were unable to determine significant differences in the characteristics between the cases (Table 3) that might have a pragmatic clinical role to guide diagnosis. The TST was negative in some of our children with confirmed TB, which is not unexpected as our children were severely malnourished. Expectedly, a history of contact was more common in children with confirmed TB; however, even this diagnostic clue was surprisingly uncommon. The stigma associated with TB in our context might have affected the validity of this information. Additionally, this history may not be available for children that live in extremely overcrowded slums as family members may not know who has transmitted TB to their child. Therefore, assessment of clinical or epidemiological risk factors for TB in our children may not be reliable. Features such as malnutrition and CXR abnormalities were part of the entry criteria, and TST positivity played a major influence in the clinical diagnosis of TB in our study. It is thus possible that our study over-diagnosed TB in the “non-confirmed” group while being under-diagnosed in the “not TB” group. Nonetheless, one third of all cases was microbiologically confirmed, and “typical” features of TB such as persistent (>2 weeks) cough was uncommon in all groups. This study highlights the need to consider the diagnosis of TB in all severely malnourished children from TB endemic settings.

Our study children tolerated the procedure of sputum induction, which also is consistent with previous observations [31], [32]. We did not observe a difference in yield between sputum sampling methods but our sample size was smaller than previous studies that have observed a significantly higher yield from induced sputum [18]. The yield from culture and Xpert MTB/RIF assay increases by taking two samples of sputum by each technique compared to one [11], [12], [13]. However, the use of a second induced sputum specimen increases the cost [33] and inconveniences to the patients. However, in young children with a low yield due to paucibacillary disease and difficulty in obtaining sputum, a second test may be worthwhile if the first test is negative [12].

In conclusion, the results of our study indicates that TB is common in severely malnourished children presenting with pneumonia in a TB endemic setting such as Bangladesh, and that Xpert MTB/RIF assay improved diagnosis in this set up. The high inpatient and post-discharge mortality in these children highlights the need for a better understanding of the preventable and treatable causes of infection in children with severe malnutrition.

## References

[1] Liu L, Johnson HL, Cousens S, Perin J, Scott S, et al (2012) Global, regional, and national causes of child mortality: an updated systematic analysis for 2010 with time trends since 2000. Lancet 379: 2151–2161. 22579125 10.1016/S0140-6736(12)60560-1

[2] Chisti MJ, Ahmed T, Faruque AS, Abdus Salam M (2010) Clinical and laboratory features of radiologic pneumonia in severely malnourished infants attending an urban diarrhea treatment center in Bangladesh. Pediatr Infect Dis J 29: 174–177. 19927041 10.1097/INF.0b013e3181b9a4d5

[3] Chisti MJ, Tebruegge M, La Vincente S, Graham SM, Duke T (2009) Pneumonia in severely malnourished children in developing countries - mortality risk, aetiology and validity of WHO clinical signs: a systematic review. Trop Med Int Health 14: 1173–1189. 19772545 10.1111/j.1365-3156.2009.02364.x

[4] Jaganath D, Mupere E (2012) Childhood tuberculosis and malnutrition. J Infect Dis 206: 1809–1815. 23033147 10.1093/infdis/jis608 PMC3502375

[5] Chisti MJ, Ahmed T, Pietroni MA, Faruque AS, Ashraf H, et al (2013) Pulmonary tuberculosis in severely-malnourished or HIV-infected children with pneumonia: a review. J Health Popul Nutr 31: 308–313. 24288943 10.3329/jhpn.v31i3.16516 PMC3805879

[6] Global Tuberculosis Report 2013. World Health Organisation, Geneva 2013.

[7] McNally LM, Jeena PM, Gajee K, Thula SA, Sturm AW, et al (2007) Effect of age, polymicrobial disease, and maternal HIV status on treatment response and cause of severe pneumonia in South African children: a prospective descriptive study. Lancet 369: 1440–1451. 17467514 10.1016/S0140-6736(07)60670-9

[8] Punia RS, Mundi I, Mohan H, Chavli KH, Harish D (2012) Tuberculosis prevalence at autopsy: a study from North India. Trop Doct 42: 46–47. 22290110 10.1258/td.2011.110314

[9] Nantongo JM, Wobudeya E, Mupere E, Joloba M, Ssengooba W, et al (2013) High incidence of pulmonary tuberculosis in children admitted with severe pneumonia in Uganda. BMC Pediatr 13: 16. 23368791 10.1186/1471-2431-13-16 PMC3584903

[10] Bates M, Mudenda V, Mwaba P, Zumla A (2013) Deaths due to respiratory tract infections in Africa: a review of autopsy studies. Curr Opin Pulm Med 19: 229–237. 23429099 10.1097/MCP.0b013e32835f4fe4

[11] Rachow A, Clowes P, Saathoff E, Mtafya B, Michael E, et al (2012) Increased and expedited case detection by Xpert MTB/RIF assay in childhood tuberculosis: a prospective cohort study. Clin Infect Dis 54: 1388–1396. 22474220 10.1093/cid/cis190

[12] Nicol MP, Workman L, Isaacs W, Munro J, Black F, et al (2011) Accuracy of the Xpert MTB/RIF test for the diagnosis of pulmonary tuberculosis in children admitted to hospital in Cape Town, South Africa: a descriptive study. Lancet Infect Dis 11: 819–824. 21764384 10.1016/S1473-3099(11)70167-0 PMC4202386

[13] Bates M, O’Grady J, Maeurer M, Tembo J, Chilukutu L, et al.. (2012) Assessment of the Xpert MTB/RIF assay for diagnosis of tuberculosis with gastric lavage aspirates in children in sub-Saharan Africa: a prospective descriptive study. Lancet Infect Dis. 10.1016/S1473-3099(12)70245-1 23134697

[14] Nhu NT, Ha DT, Anh ND, Thu do DA, Duong TN, et al (2013) Evaluation of Xpert MTB/RIF and MODS assay for the diagnosis of pediatric tuberculosis. BMC Infect Dis 13: 31. 23343418 10.1186/1471-2334-13-31 PMC3562258

[15] The state of the World’s Children 2013. United Nations Children’s Fund (UNICEF), 2013.

[16] Ahmed T, Ali M, Ullah MM, Choudhury IA, Haque ME, et al (1999) Mortality in severely malnourished children with diarrhoea and use of a standardised management protocol. Lancet 353: 1919–1922. 10371570 10.1016/S0140-6736(98)07499-6

[17] WHO (2005) Pocket book for hospital care of children: guidelines for the management of common illness with limited resources. World Health Organization, Geneva. 24006557

[18] Zar HJ, Hanslo D, Apolles P, Swingler G, Hussey G (2005) Induced sputum versus gastric lavage for microbiological confirmation of pulmonary tuberculosis in infants and young children: a prospective study. Lancet 365: 130–134. 15639294 10.1016/S0140-6736(05)17702-2

[19] World Health Organization (2006) Guidance for national tuberculosis programmes on the management of tuberculosis in children. Geneva. 24999516

[20] Grant LR, Hammitt LL, Murdoch DR, O’Brien KL, Scott JA (2012) Procedures for collection of induced sputum specimens from children. Clin Infect Dis 54 Suppl 2 S140–145. 22403228 10.1093/cid/cir1069 PMC3297553

[21] National guideline for the management of tuberculosis in children (2012) National Tuberculosis Control Programme, Directorate General of Health Services, Ministry of Health and Family Welfare, Dhaka, Bangladesh.

[22] Banu S, Mahmud AM, Rahman MT, Hossain A, Uddin MK, et al (2012) Multidrug-resistant tuberculosis in admitted patients at a tertiary referral hospital of Bangladesh. PLoS One 7: e40545. 22808189 10.1371/journal.pone.0040545 PMC3394739

[23] Kim BJ, Lee SH, Lyu MA, Kim SJ, Bai GH, et al (1999) Identification of mycobacterial species by comparative sequence analysis of the RNA polymerase gene (rpoB). J Clin Microbiol 37: 1714–1720. 10325313 10.1128/jcm.37.6.1714-1720.1999 PMC84932

[24] Chisti MJ, Saha S, Roy CN, Salam MA (2010) Predictors of bacteremia in infants with diarrhea and systemic inflammatory response syndrome attending an urban diarrheal treatment center in a developing country. Pediatr Crit Care Med 11: 92–97. 19593244 10.1097/PCC.0b013e3181b063e1

[25] Cherian T, Mulholland EK, Carlin JB, Ostensen H, Amin R, et al (2005) Standardized interpretation of paediatric chest radiographs for the diagnosis of pneumonia in epidemiological studies. Bull World Health Organ 83: 353–359. 15976876 PMC2626240

[26] Carrol ED, Mankhambo LA, Guiver M, Banda DL, Denis B, et al. PCR improves diagnostic yield from lung aspiration in Malawian children with radiologically confirmed pneumonia. PLoS One 6: e21042. 10.1371/journal.pone.0021042 PMC3114850 21695128

[27] Azim T, Rahman M, Alam MS, Chowdhury IA, Khan R, et al (2008) Bangladesh moves from being a low-prevalence nation for HIV to one with a concentrated epidemic in injecting drug users. Int J STD AIDS 19: 327–331. 18482963 10.1258/ijsa.2007.007269

[28] Waterlow JC (1997) Protein-energy malnutrition: the nature and extent of the problem. Clin Nutr 16 Suppl 1 3–9. 16844615 10.1016/s0261-5614(97)80043-x

[29] Berkowitz FE (1992) Infections in children with severe protein-energy malnutrition. Pediatr Infect Dis J 11: 750–759. 1448316 10.1097/00006454-199209000-00015

[30] Ongoing. Cotrimoxazole Prophylaxis in Severely Malnourished Children: a randomized, double blind, placebo-controlled trial. ClinicalTrials.gov Identifier: NCT00934492.

[31] Shata AM, Coulter JB, Parry CM, Ching’ani G, Broadhead RL, et al (1996) Sputum induction for the diagnosis of tuberculosis. Arch Dis Child 74: 535–537. 8758132 10.1136/adc.74.6.535 PMC1511555

[32] Wiersma HE, Van Aalderen WM, Hoekstra MO (2000) Sputum induction for the diagnosis of pulmonary tuberculosis. Arch Dis Child 83: 276. 10.1136/adc.83.3.276g PMC1718453 10991758

[33] Tuberculosis diagnostics: Automated DNA test. World Health Organization, Geneva, 2011. Available: http://www.who.int/tb/features_archive/xpert_factsheet.pdf. Accessed 2013 Jun 21.

